# Risk Factors for Human Infection with Puumala Virus, Southwestern Germany

**DOI:** 10.3201/eid1507.081413

**Published:** 2009-07

**Authors:** Anne Caroline Schwarz, Ulrich Ranft, Isolde Piechotowski, James E. Childs, Stefan O. Brockmann

**Affiliations:** Bernhard-Nocht-Institute for Tropical Medicine, Hamburg, Germany (A.C. Schwarz); Institut für umweltmedizinische Forschung, Düsseldorf, Germany (U. Ranft); Baden-Württemberg State Health Office, Stuttgart, Germany (I. Piechotowski, S.O. Brockmann); Yale University School of Medicine, New Haven, Connecticut, USA (J.E. Childs)

**Keywords:** Hantavirus, climate change, Puumala virus, bank vole, modeling, viruses, zoonoses, Germany, research

## Abstract

Puumala virus, which causes nephropathia epidemica (NE), is the most prevalent hantavirus in Germany; bank voles serve as the main reservoir. During 2001–2007, most NE cases reported from Germany occurred in the southwestern state of Baden-Württemberg. We investigated the influence of bank vole habitats (beech forest, seed plants), vole food supply (beechnut mast), climate factors (winter and spring temperatures), and human population density on spatial and temporal occurrence of NE cases in Baden-Württemberg. Using Poisson-regression analyses, we found that all these factors influenced disease incidence. Furthermore, an independent trend of increasing incidence predicted that incidence will nearly double each year. The regression model explained 75% of the annual variation in NE incidence. The results suggest that environmental drivers lead to increasing incidence of NE infections in the southern part or even other parts of Germany.

## Abstract 2

Risk factors are available bank vole habitat, abundant vole food supply, high human population density, and warmer climate.

Hantaviruses (family *Bunyaviridae*) are the etiologic agents of 2 distinct clinical syndromes: hemorrhagic fever with renal syndrome and hantavirus pulmonary syndrome (*1*,*2*). The former occurs in Asia and Europe; the latter, in the Americas (North, Central, and South) (*3*). Of the ≈30 hantaviruses described, approximately half are of clinical relevance (*4*–*6*). In Germany, the most common hantaviral disease is nephropathia epidemica (NE), which is associated with Puumala virus (PUUV) infection (*7*,*8*). The primary rodent reservoir for PUUV is the bank vole (*Myodes glareolus,* formerly *Clethrionomys glareolus*), which in Europe extends south from Scandinavia to Italy and Spain (*9*).

NE is considered a mild form of hemorrhagic fever with renal syndrome; mortality rate is <1%. After an incubation period of 2–4 weeks, disease onset is abrupt; major signs and symptoms are fever, headache, back pain, abdominal pain, and other gastrointestinal involvement. Occasionally, acute renal failure develops and the patient may require hemodialysis.

Transmission of hantaviruses from rodents to humans is believed to occur through inhalation of aerosols contaminated by virus shed in excreta, saliva, and urine of infected animals (*10*). Human-to-human transmission of hantaviruses is rare, although investigations in Argentina implicated Andes virus with this type of infection (*11*).

One hypothesis suggests that the risk for human infection with hantaviruses increases with the population size of the reservoir host species, which can be driven to high levels in response to events that enhance host survival, promote early breeding, and increase the food supply (*12*). Such events may result from climatic perturbations, such as the El Niño Southern Oscillation, which increases precipitation and results in unusually mild winters. It has been hypothesized that *Peromyscus maniculatus* rodents, the reservoir host for Sin Nombre virus (SNV), increased as a result of the El Niño Southern Oscillation (*13*). In Europe, researchers recently demonstrated a positive relationship between tree seed production, milder climate, and NE incidence (*14*). The availability of suitable habitat for rodents is also a key factor to consider when assessing the risk for hantavirus transmission.

Although environmental factors influence the availability and quality of suitable rodent habitat and resources, risk for hantaviral disease transmission to humans also depends on human proximity, behavior, and land-use patterns. Furthermore, peridomestic exposure might increase as rodent reservoirs increase and rodents disperse to buildings. Therefore, investigations assessing risk for hantaviral infection must evaluate factors influencing the reservoir host population, the human population at risk, and potential factors driving their interaction.

Since NE became a notifiable disease in Germany in 2001, most cases have been reported from the state of Baden-Württemberg, in southwestern Germany (*15*), where the number of cases varied from a minimum of 17 in 2006 to a maximum of 1,077 in 2007. Our objective was to investigate the association between NE incidence in southwestern Germany and environmental factors that potentially influence the PUUV reservoir abundance and, hence, the risk of acquiring NE. We used statistical modeling to assess the influence of vole habitat (presence and quality), annual variation in vole food source, variation in climatic conditions, and the human population at risk on the annual NE incidence during a 7-year period. Specifically, we investigated whether NE incidence is positively associated with availability of suitable rodent habitat and supply of food, human population density, and a rise in winter or spring temperatures above long-term averages.

## Materials and Methods

### Incidence of NE

The annual total of patients with symptomatic, laboratory-confirmed cases of NE reported from the state of Baden-Württemberg was provided by the German surveillance system for infectious diseases, which covers all 44 districts of Baden-Württemberg, for 2001–2007. Laboratory diagnosis was based either on detection of viral RNA by reverse transcription–PCR or on detection of immunoglobulin (Ig) M or a marked rise of antihantavirus IgG (*15*). Details of the German notification system are provided by Faensen et al. (*16*), and detailed information about the incidence of NE in Germany can be obtained from Piechotowski et al. (*15*). We excluded from analysis those patients who reported recent travel. We obtained human population density by district from the Statistical Office in Baden-Württemberg (*17*). Because the population of Baden-Württemberg in the respective districts did not change substantially during 2001–2007, we used census data for 2006 to calculate incidence rates (no. cases/100,000 inhabitants) for each study year.

### Bank Vole Factors, Habitat, and Beechnut Mast

Factors considered to favor bank vole habitat were obtained from the Forest Research Institute Baden-Württemberg, Department of Biometry and Information Science, from forest inventories conducted during 2001–2002 (*18*). We assessed percentage of land cover for 5 covariates that have been indicated as preferred habitat for bank voles in Europe (*19*,*20*): beech forests, seed plants, bilberries, dwarf shrubs, and blackberries. For oak forests, also a preferred habitat, no data were available. Data for beech forest cover were provided as hectares per district and converted to proportion coverage per district area. For each of the 4 remaining habitat areas by district, data were provided as area variables in 3 categories of coverage: rare (1%–10%), frequent (>10%–50%), and common (>50%). To estimate the availability of the respective habitat in each district, we calculated a weighted sum of the 3 coverage areas by using 0.01, 0.1, and 0.5 as weights for the rare, frequent, and common areas, respectively, and then converted each sum into percentage coverage per district area. For each district, the 5 habitat variables were considered constant during the study period.

We obtained data on annual mast production of beechnuts by district, starting and ending 1 year earlier than the study (2000–2006). Data came from the Ministry for Agriculture Baden-Württemberg and the Public Forest Administration Baden-Württemberg, for which foresters conduct annual counts of beechnuts under beech trees (plot counts) (*21*). The beechnut mast in the preceding year was used as a potential determinant of the NE incidence because beechnut supply in the fall may influence winter vole survival and, consequently, vole population the following year. Beechnut mast data were stratified into 3 classes: good/excellent crop if 40%–100% of trees produced mast, medium if 10%–39%, and poor if 0%–9%.

### Climate Factors

Deviations of the monthly temperature for 2000–2007 were referenced against the perennial average for 1961–1990, provided by the German Meteorological Service (*22*). The station that geographically best represented a respective district was selected from the network of 576 meteorology stations covering Germany. Only the temperature deviations of the winter and early spring months (December–March) were included because mild winters and springs were hypothesized to enhance survival rates of rodents and produce food resources earlier. For each year and district we modeled mean values of temperature deviations for winter (December of the preceding year and January of the same year as NE incidence data) and spring (February and March of the same year).

### Statistical Modeling

We modeled associations between potential risk factors and incidence rates of NE during 2001–2007 by multivariate Poisson regression, using the SAS program GENMOD (version 9.1, SAS Institute Inc., Cary, NC, USA). In Poisson regression, we set the logarithm of the expected annual incidence per district and year equal to a linear term of potential determinants: habitat variables, climate factors, human population density, and year of investigation. The number of NE cases per year and district were approximately Poisson distributed. To allow for overdispersion, we introduced a scale parameter in the regression modeling. To account for nonlinearity, the variable “year of investigation” was represented in the regression model by 6 dichotomous variables. All other independent variables were considered continuous and were scaled by units as per 5% increase in district-area coverage by beech forest and seed plants, per increase in human population density of 500 inhabitants per square kilometer, per 1°C change in winter and spring temperature above the long-term average, and per unit step of beechnut mast (good/excellent, medium, poor). The covariates in the Poisson regression model were examined for 2-way interactions, but none could be confirmed.

The criterion for inclusion of a determinant in the final regression model was set at a significance level of p<0.05. For a measure of association between a determinant and NE incidence, the risk ratio (RR) was calculated by using the respective estimated regression parameter of the Poisson regression model and, therefore, adjusted for all other determinants included in the regression model. All estimates of RR were complemented by a 95% confidence interval (CI) and p value. The pseudo-R-squared (R^2^) was provided as a measure of overall goodness-of-fit of the regression model.

## Results

**Figure 1:**
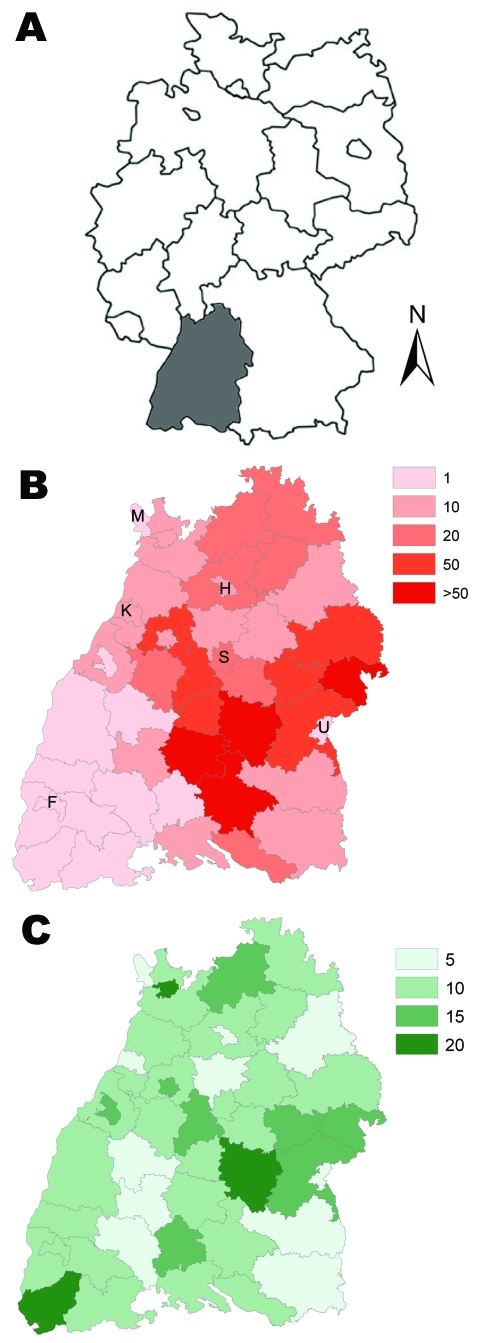
A) Map of Germany showing location of Baden-Württemberg region (gray shading). B) Cumulative incidence (per 100,000 population) of nephropathia epidemica, Baden-Württemberg, Germany, 2001–2007. Letters indicate major cities: F, Freiburg; H, Heilbronn; K, Karlsruhe; M, Mannheim; S, Stuttgart; U, Ulm. C) Percentage cover of beech forest.

A total of 1,540 NE cases were reported from the study area during 2001–2007 and were included in the analysis. The median values of NE incidence in all districts varied from 0 (in 2002, 2003, and 2006) to 2.28 in 2007 (Table 1); the lowest maximum incidence rate was 1.16 in 2006, and the highest maximum incidence rate was 90.19 in 2007. Mapping of the cumulative district NE incidence in Baden-Württemberg indicated that the districts reporting the highest incidence rate for cases clustered within the southeastern Swabian Alb region during 2001–2007 (Figure 1, panel B).

**Table 1:** Nephropathia epidemica in Baden-Württemberg, Germany, by year, 2001–2007*

Year	No. cases†	Incidence/100,000 population				Temperature, °C								*Beechnut crop¶*		
						Winter‡				Spring§						
		Min	Med	Max		Min	Med	Max		Min	Med	Max		Min	Med	*Max*
2001	37	0.0	0.25	4.5		2.0	2.5	3.5		2.0	2.5	2.5		0	0	*1*
2002	140	0.0	0.0	11.18		−0.5	0.5	0.0		3.0	3.5	3.5		1	2	2
2003	55	0.0	0.0	3.33		0.5	1.0	1.5		−0.5	0.0	1.0		1	1	2
2004	109	0.0	0.27	6.01		0.5	0.5	1.5		0.0	1.0	1.5		1	1	2
2005	105	0.0	0.70	4.07		0.0	−0.5	1.0		−1.5	−1.0	−0.5		1	1	2
2006	17	0.0	0.0	1.16		−2.0	−1.5	−1.0		−2.0	−1.5	−1.0		0	0	0
2007	1,077	0.0	2.28	90.19		3.0	2.5	3.0		2.0	2.5	3.0		2	2	*2*
*Min, minimum; med, median; max, maximum. †Total no. cases for all districts in Baden-Württemberg. ‡Temperature deviation from the long-term average for December of the previous year and January of the actual year. §Temperature deviation from the long-term average for February and March. ¶Beechnut crop of the preceding year in 3 categories: 0, poor crop (0%–9% of a mast year); 1, medium crop (10%–39% of a mast year); 2, good/excellent crop (40%–100% of a mast year).																

### Time-dependent Factors

The annual percentage of statewide beechnut mast varied by year and district from a crop failure (0%–9% of optimum mast) in 2005 to good/excellent (40%–100% of optimum mast) mast in 2001 and 2006 (Table 1). Winter temperatures exceeded long-term averages in 2001, 2003, 2004, and 2007. The maximum winter temperature deviation occurred in 2007; median deviation was +3.5°C and maximum was +4.5°C. Winter temperatures were below the long-term averages in 2002, 2005, and 2006 (range of deviation –0.5°C to –1.5°C). Mean spring temperature exceeded the long-term average for all years except 2005 and 2006 (Table 1).

### Time-independent Factors

Maximum beech forest cover within a district (17.7%) was found in the region of the Swabian Alb (Table 2; Figure 1, panel C); lowest beech forest cover was found in the eastern and middle regions, as well as in districts containing the major cities of Baden-Württemberg. Maximum seed plant cover (11.3%) was found in the southerly and centrally located districts of Baden-Württemberg; the lowest cover (1.3%) was associated with the northerly districts and in the districts containing the major cities. The median values of the land cover variables of dwarf shrubs, bilberry, and blackberry were <2%; values of ≈10% were restricted to a few districts (Table 2). As the percentages of land cover in bilberry and dwarf shrubs were closely correlated across districts (r ≈ 1), only 1 of these variables (bilberry) was included in the regression modeling. Human population density ranged from 104 inhabitants/km^2^ in rural districts to 2,864 inhabitants/km^2^ in the state capital of Stuttgart (Table 2; Figure 1, panel B). Most districts (80%) contained <1,000 inhabitants/km^2^.

**Table 2:** Bank vole habitat and human population density per district, Baden-Württemberg, Germany, 2001–2007

Density	Habitat cover, % total district area					*Human population density/km^2^ district area*
	Beech forest	Seed plants	Dwarf shrubs	Bilberry	Blackberry	
Minimum	1.6	1.3	0.0	0.0	0.2	*104*
Median	8.1	3.9	0.2	0.2	1.8	*323*
*Maximum*	*17.7*	*11.3*	*10.6*	*10.3*	*6.2*	*2,864*

### Poisson Regression Analysis

**Figure 2:**
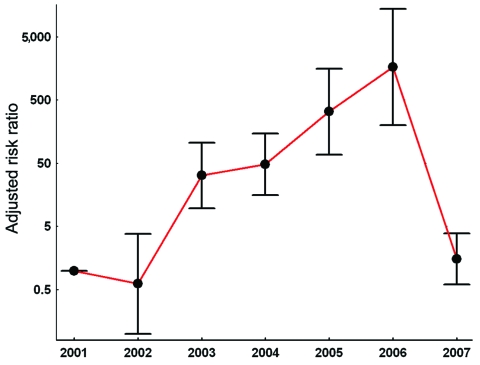
Influence of the year of investigation on the incidence of nephropathia epidemica, indicated by adjusted risk ratios estimated by Poisson regression analysis. 2001 is reference year. Error bars indicate 95% confidence intervals. For controlling covariates, see Table 3.

For the final Poisson regression model, only the blackberry and bilberry variables did not pass the inclusion criterion of a positive association with the annual incidence of NE during 2001–2007 and a significance level of p<0.05 (Table 3; Figure 2). The final model explained 75% of the variation of NE incidence (R^2^ = 0.75).

**Table 3:** Influence of determinants on incidence of nephropathia epidemica, Baden-Württemberg, Germany, 2001–2007*

Determinant	Risk ratio†	95% Confidence interval	p value‡
Supply of beechnut§	2.86	1.81–4.50	<0.0001
Cover of beech forest¶	1.94	1.69–2.22	<0.0001
Cover of seed plant¶	2.80	2.31–3.40	<0.0001
Winter temperature deviation#	1.70	1.11–2.61	0.0156
Spring temperature deviation#	4.49	2.86–7.06	<0.0001
Human population density**	1.12	1.01–1.23	0.0265
Year of investigation††	NA	NA	<0.0001

*Multivariate Poisson regression analysis; NA, not applicable. †Mutually adjusted. ‡Likelihood ratio Test §Good/excellent crop year referenced to a medium crop year. ¶Unit = 5%. #Unit = 1°C. **Unit = 500/km^2^. ††Dichotomized (Figure 2).

The variables showing annual variation, specifically beechnut mast and spring temperature above the long-term mean, were strong independent predictors of NE. The occurrence of a good/excellent beechnut mast in the previous year increased risk for NE (RR 2.86, 95% CI 1.81–4.50) relative to medium crop (Table 3). An increase of 1°C in spring (February/March) above the long-term average resulted in RR of 4.49 (95% CI 2.86–7.06). The influence of winter (December/January) temperature was less strong, but it was still a significant factor in NE incidence; RR 1.70 (95% CI 1.11–2.61).

The association between NE incidence and year of investigation indicated that incidence increased exponentially during 2002–2006, when all other risk factors were controlled for and 2001 was used as a reference. However, this trend was not apparent in 2007. In 2007, extreme winter and spring temperatures relative to the long-term average, coupled with the best beechnut mast observed during the entire study interval, potentially masked or overwhelmed the temporal trend toward increasing NE incidence in the regression modeling. To check stability of the observed time trend, we repeated the regression analysis with the investigation year 2007 excluded. Again, a highly significant time trend, which indicated a doubling of the NE incidence per year (RR 2.02, 95% CI 1.43–2.83), was observed. Habitat and climate factors as well as human population density had somewhat lower, but still highly significant, estimates of RR (data not shown). When the estimated 2001–2006 regression model was used for prediction, the incidence in 2007 was considerably underestimated. This result underlines the importance of the time-dependent habitat and climate factors for the PUUV reservoir.

As notification of NE cases became a requirement in Germany in 2001, underreporting could have occurred in the first year of investigation (2001). However, exclusion of 2001 in the regression analysis did not change the significance and magnitude of the associations of the included risk factors with the NE incidence (data not shown).

Among the time-invariant determinants, 2 of the land-cover parameters—percentage of beech forest (Figure 1, panel C) and seed plant cover—exhibited a major effect. For each 5% increase in coverage per district, risk for NE approximately doubled (beech forest, RR 1.94, 95% CI 1.69–2.22; seed plants, RR 2.80, 95% CI 2.31–3.40; Table 3). A unit increase in human population density of 500 inhabitants/km^2^ (about the median population density of the 44 districts) increased incidence of NE by ≈12% (RR 1.12, 95% CI 1.01–1.23).

## Discussion

The analysis of NE in the state of Baden-Württemberg during 2001–2007 clearly indicated a strong association of variables reflecting preferred bank vole habitat and abundance of a major food resource (beechnut mast), in addition to relative mild spring and winter temperatures, with the spatial and temporal incidence of PUUV infection. Furthermore, human population density was a weak but statistically significant determinant of NE incidence. The results also indicate that risk of acquiring NE increased over the 7-year study period, possibly forecasting a trend of increasing incidence of PUUV infection in southern Germany. The estimated Poisson regression model accounted for 75% of the spatial and temporal variation in NE incidence during 2001−2007.

The direct influence of mild winter and spring temperatures on NE incidence cannot be interpreted independently from other annual fluctuations, such as the quality of beechnut mast. As an example, winter and spring temperatures during 2000–2001 were as much as +3.5°C and +2.5°C above the long-term average, but the beech mast in 2000 was one of the lowest reported, and NE incidence was low in 2001 (median 0.25). When winter and spring temperatures above long-term average were coincident with a good/excellent beech mast, as in 2006–2007, incidence of reported NE in 2007 was the highest recorded (median 2.28) (Table 1).

After mild winter and spring conditions, and when supplied with a rich food resource from the previous fall, rodent populations likely reach higher densities through a combination of increased overwinter survival rate and earlier onset of breeding. Increased rodent abundance or density could increase risk for human contact with a PUUV-infected rodent and, thus, increase risk for NE. In the Great Basin desert area of the western United States, higher population densities of the rodent reservoir host for SNV and, consequently, increased incidence of hantavirus pulmonary syndrome have also been hypothesized to be driven by weather anomalies, which result in increased food sources after milder winters with increased rainfall (*23*,*24*). Mild winter was also hypothesized as a factor leading to an outbreak of PUUV infection 2007 in northern Sweden (*25*). However, in northern European locations where NE is endemic, different factors may contribute or drive the risk for human infection by PUUV. In Sweden, most human NE cases occur in late autumn or early winter and are believed to occur when rodents seeking to avoid harsh winter conditions move into human dwellings for shelter (*26*). In Germany, most cases occur in early summer (*15*), possibly when recreational and occupational activities bring people into bank voles’ environments. Outdoor activities may also contribute to human peridomestic exposure to PUUV. A case–control study conducted in 2007 in Baden-Württemberg demonstrated that visiting or cleaning human shelters in the forest, among other factors, increased risk of acquiring the disease (*27*).

The influence of time-independent determinants also highlights patterns influencing risk for NE. The strong associations of beech forest and seed plant cover with NE incidence support the hypothesis that indices of preferred bank vole habitat, where bank vole populations reach their highest density (*28*,*29*), are associated with elevated levels of PUUV transmission. The area with the highest percentages of beech forest and seed plant coincided with the Swabian Alb region, to which NE is highly endemic and the highest incidence rate (90.19) has been reported. In contrast, NE is rarely reported from the Black Forest region, which contains the highest elevations of Baden-Württemberg (as high as 1,500 m) and has a primary land cover of coniferous forest, a habitat not preferred by bank voles (*28*,*29*).

Other studies have also demonstrated a strong link between habitat indices for a rodent species serving as a reservoir host for a hantavirus and increasing risk for human disease. In Sweden, increasing abundance of bank voles and increasing numbers of PUUV-infected voles were associated with an environment composed of old-growth moist forests (*30*). In the Great Basin desert of the United States, deciduous or mixed forest, grasslands, and pinyon–juniper woodlands are associated with varying risk for hantavirus pulmonary syndrome (*24*,*31*).

The potential for human interaction with bank voles being a risk factor for NE incidence was suggested by the positive and significant association of NE incidence with human population density. Density served as a surrogate measure of actual human–rodent contact, which occurs when animals enter buildings or when humans participate in outdoor activities. Other surveillance-based studies of a zoonotic virus have linked human abundance with risk for disease exposure (*32*).

Additional biologic and environmental factors may be associated with the risk for hantavirus transmission to humans or may influence rodent abundance. Annual variation in precipitation has been suggested (*23*,*24*). Geo-ecologic variables, such as elevation, slope, or geology and soil type, have been linked to the risk for hantavirus infection in the US Great Basin (*24*). Indeed, the geologic substrate provides some measure of the dominant land-cover classes of vegetation. Beech trees prefer karst, with the soil types rendzina and cambisol, which is mostly found on the Swabian Alb, where most NE cases occurred. Other studies have shown that increased predators decrease populations of bank voles (*33*,*34*). An effect of predators on community composition and species abundance has been suggested for other vector-borne diseases, including hantaviruses (*35*,*36*).

Precise information on where NE was acquired was unavailable and therefore precluded our ability to analyze these site-specific factors. Therefore, a case–control study was initiated to provide a more detailed analyses of site-specific risk factors and to collect better information on presumed locations where transmission of PUUV occurred (*27*).

Surveillance data are subject to bias (*37*). Because the designation of NE as a notifiable disease in Germany is relatively recent, spatial and temporal modeling of surveillance data enhance the usefulness of these data by predicting disease trends and potentially assessing the quality of disease reporting (*37*).

Our results suggest that global climate anomalies, or the increasing trend toward warmer annual temperatures, could have a considerable effect on NE in Germany. Many regions in the world appear to be at increased risk for outbreaks of vector-borne and zoonotic diseases such as Rift Valley fever, West Nile fever (*38*), and NE (*15*,*16*,*25*,*27*). The effect of vector-borne and zoonotic diseases is dynamic and strongly linked to environmental drivers in addition to changes in human demographics and behavior. The current dispersion of NE in Germany and the increasing incidence, especially in the state of Baden-Württemberg, pose a risk to public health and require monitoring. Because no vaccine against NE is available and the potential costs of medical care associated with severe disease can be high, public health recommendations for reducing the risk for PUUV infection should be further promoted and evaluated, as has occurred in the United States (*39*). Analyses such as ours can help focus future studies and enhance surveillance efforts and evaluation of prevention measures by predicting where humans are at greatest risk for NE.
